# The utility of HbA1c combined with haematocrit for early screening of gestational diabetes mellitus

**DOI:** 10.1186/s13098-018-0314-9

**Published:** 2018-03-05

**Authors:** Kui Wu, Yan Cheng, Tingting Li, Ziwen Ma, Junxiu Liu, Qingying Zhang, Haidong Cheng

**Affiliations:** 1 Obstetrics and Gynecology Hospital, Fudan University, 128 Shenyang Road, Shanghai, 200090 People’s Republic of China; 2 Shanghai Key Laboratory of Female Reproductive Endocrine Related Diseases, Shanghai, 200011 China

**Keywords:** Glycated haemoglobin A1c (HbA1c), Haematocrit (HCT), Gestational diabetes mellitus (GDM)

## Abstract

**Aims:**

To evaluate the utility of glycated haemoglobin A1c (HbA1c) alone and in combination with haematocrit (HCT) for screening gestational diabetes mellitus (GDM) between 12–16 gestational weeks.

**Methods:**

This prospective study was carried out in the Obstetrics and Gynaecology Hospital of Fudan University from November 2014 to February 2015. In total, 690 pregnant women between 20 and 35 years old were included in this study. All subjects received a routine blood examination for HbA1c and HCT at 12–16 gestational weeks (gw) and a 75-g oral glucose tolerance test at 24–28 gw. Threshold values for the diagnosis of GDM were a plasma glucose concentration of 5.1 mmol/L after fasting, 10.0 mmol/L at 60 min, and 8.5 mmol/L at 120 min. Receiver operating characteristic curves were used to evaluate the diagnostic performance of HbA1c with or without HCT.

**Results:**

One hundred seven women were diagnosis with GDM at 24–28 gw. An HbA1c cutoff value < 4.55% at 12–16 gw showed adequate sensitivity to exclude GDM (85.0%) but low specificity (17.3%), while an HbA1c cutoff value ≥ 5.25% presented adequate specificity (96.6%) but low sensitivity (13.3%) in diagnosing GDM. The area under the receiver operating characteristic curve for HbA1c (12–16 gw) detection of GDM was 0.563 (95% confidence interval [CI], 0.50–0.625). When combined HbA1c with HCT ( > 38.8%) for the screening of GDM, the area under the receiver operating characteristic curve was 0.604 (95% [CI] 0.509, 0.701).

**Conclusions:**

Whether the adoption of HbA1c as a screening test for GDM would benefit pregnant women remains to be determined. However, combining HbA1c with HCT for the screening of GDM may be a useful tool to predict GDM.

## Introduction

Gestational diabetes mellitus (GDM) is a pregnancy complication that is linked with insulin resistance and increases the risk of macrosomia and perinatal morbidity and mortality for the foetus, while presaging a long-term risk of developing type 2 diabetes for the mother [1–3]. The diagnosis of GDM is performed with a 75-g oral glucose tolerance test (OGTT) at 24–28 weeks gestation for all pregnant women [4]. However, screening methods of GDM before the usual window of 24–28 weeks’ gestation are lacking. Some clinical guidelines suggest that women who have risk factors of GDM should perform a 75-g OGTT test earlier than 24–28 gestational weeks. However, OGTT is costly, time-consuming, and labor intensive, and has low reproducibility which can add to the confusion and uncertainty in confirming a diabetes diagnoses [5]. The accuracy of OGTT may be reduced by patient non-adherence to fasting and/or the use of certain medications [6]. There have not been sufficient studies performed to know whether there is a benefit of testing GDM before 24–28 weeks’ gestation. Therefore, alternative strategies that do not require more than a single blood draw may increase the rate of GDM testing [7].

Glycated haemoglobin (HbA1c) is the product of an irreversible non-enzymatic binding of glucose to plasma proteins, specifically haemoglobin (Hb). The mean plasma glucose over the erythrocyte life span is correlated with a degree of glycosylation. It is a single, non-fasting blood test and reflects the glucose levels over the previous 4–8 weeks. Therefore, HbA1c measurement has become an attractive option, as it is easily added to the routine early pregnancy laboratory tests (first antenatal blood draws) in the non-fasting patient. HbA1c levels have been proposed as a diagnostic tool for identifying patients with undiagnosed diabetes or a risk of developing diabetes [8]. However, HbA1c measurement had not been sufficiently standardized and HbA1c is affected by red blood cell turnover in addition to plasma glucose [9]. So, the usefulness of glycated haemoglobin (HbA1c) as a tool to assess glycaemic status in pregnant women remains controversial. There are no recommendations available for the use of HbA1c as a diagnostic tool for GDM [10–12].

The relationship between a high haematocrit (HCT) level and insulin resistance has been reported outside pregnancy [13, 14], and a high HCT at the first visit for prenatal care was an independent predictor of GDM in a multi-ethnic Asian population [15]. Although the pathogenesis between HCT and the development of GDM is still not clearly understood, HCT is associated with insulin sensitivity [16].

Given that the HbA1c value reflects the average level of glycaemia over the preceding 2–3 months and the HCT was related with GDM, combining the use of the two indicators may improve the accuracy of the prediction.

Therefore, the aims of this study were to determine the optimal early pregnancy HbA1c threshold to detect GDM and whether the combination of HCT and HbA1c can be used as a tool for the screening of GDM in Chinese community settings.

## Research design and methods

### Study population

We enrolled 987 women (aged from 20 to 35 years) into the prospective cohort, in which all women were required to participate in antepartum screening between 12–16 weeks of pregnancy from November 2014 to February 2015. The institutional review committee approved this study. The final study population for the analysis consisted of 690 women. Two hundred ninety-seven women were excluded due to the following exclusion criteria: type 2 diabetes, a fasting plasma glucose > 5.6 mmol/L, which identified women with impaired fasting glucose levels or diabetes [17], alcohol consumption, cigarette smoking, haematological diseases, comorbidities or major organ dysfunction, thyroid disease history, in vitro fertilization-embryo transfer (IVF-ET), multiple pregnancies, hypertension history, and hyperemesis history. All subjects were screened for GDM using the 75-g OGTT between 24–28 gw. GDM was diagnosed by the International Association of Diabetes and Pregnancy Study Group (IADPSG) criteria.

### Laboratory measurements

The test was performed after 3 days of normal carbohydrate intake and physical activity, and venous blood samples were drawn after an overnight fast of at least 12 h, to avoid excessive blood concentrations. All the participants accepted an initial prenatal screening at 12–16 gw, which included physical examination, anthropometric measurements, biochemical measurements, and a questionnaire on health-related behaviour, such as alcohol consumption, cigarette smoking, reproductive history, menstrual history, and physical activity. Medical history and a history of prescription drug use were assessed by the examining physicians. A family history of diabetes was defined as the presence of a mother, father, sister, or brother with type 2 diabetes diagnosed by a physician. Body mass index (BMI) was used as a measure of overall obesity (kg/m^2^). After a 10-min rest in a quiet room, systolic and diastolic blood pressures were measured in the right arm using an electronic sphygmomanometer. Total cholesterol, high-density lipoprotein (HDL)-cholesterol, triglycerides, glycated albumin, Vitamin B12, and fasting blood glucose was measured by 7600 series automatic analyser (Hitachi, Tokyo, Japan). HbA1c was measured using the VARIANT II TURBO Hemoglobin Testing System (Bio-Rad, California,USA). HCTs were determined using a XN-1000i autoanalyser (Sysmex, Hyogo, Japan). When performing the 75-g OGTT, venous blood samples were taken from all the participants in the morning and were measured at fasting and at 60 and 120 min following the ingestion of glucose as previously described. The threshold values for the diagnosis of GDM were a plasma glucose concentration of 5.1 mmol/L after fasting, 10.0 mmol/L at 60 min, and 8.5 mmol/L at 120 min.

### Statistical analysis

All analyses were accomplished using SPSS 19.0 software. The Chi squared (χ^2^) test was used for categorical variables. One-way analysis of variance was used to analyse the significant differences among the characteristics of the study participants at entry, according to the HCT level. Categories of HCT were defined by the following tertiles: < 37.1, 37.1–38.8, and > 38.8%. A receiver operating characteristic (ROC) curve was used to evaluate the diagnostic performance of HbA1c. The Youden index formula is defined as J = sensitivity + specificity − 1, which is equivalent to the maximum sum of sensitivity and specificity for all the possible values of the cutoff point [18]. The level of significance was 0.05.

## Results

Baseline characteristics of all participants are shown in Table 1. Among the eligible women, 107 (15.5%) were diagnosed with GDM using OGTT according to the IADPSG criteria.

**Table 1:** Comparison clinical characters of women with and without GDM

Variable	Non-GDM (n = 583)	GDM (n = 107)	P
Age (years)	30.14 ± 3.23	31.21 ± 3.30	0.002
Body mass index (kg/m^2^)	20.72 ± 2.61	22.85 ± 2.66	< 0.001
Diastolic blood pressure (mmHg)	69.82 ± 8.64	72.76 ± 7.65	0.001
Systolic blood pressure (mmHg)	112.32 ± 11.58	117.02 ± 10.86	< 0.001
Red blood cell count (×10^12^)	4.18 ± 0.32	4.26 ± 0.30	0.01
Hemoglobin (g/L)	122.43 ± 8.87	125.82 ± 8.22	0.001
Platelet count (×10^9^)	231.62 ± 50.12	240.09 ± 53.41	0.112
Hematocrits (%)	37.11 ± 2.36	37.93 ± 2.18	0.001
Glycated albumin (%)	13.7 ± 1.16	13.32 ± 1.25	0.002
Glycated hemoglobin (%)	4.80 ± 0.29	4.89 ± 0.33	0.004
Total cholesterol (mmol/L)	4.69 ± 0.71	4.80 ± 0.69	0.116
Triglyceride (mmol/L)	1.51 ± 0.57	1.83 ± 0.70	< 0.001
HDL (mmol/L)	1.03 ± 0.17	1.06 ± 0.17	0.051
Vitamin B12 (pg/mL)	510.80 ± 177.51	477.28 ± 143.89	0.035
Folic acid (ng/mL)	16.11 ± 2.82	16.81 ± 2.21	0.016
Fasting blood glucose (mmol/L)	4.23 ± 0.30	4.42 ± 0.40	< 0.001

*HDL* high density lipoprotein

Categories of HCT were defined by the following tertiles: < 37.0, 37.1–38.8, and > 38.8%; the number of pregnant women in each category was 241, 225 and 224, respectively.

**Fig. 1:**
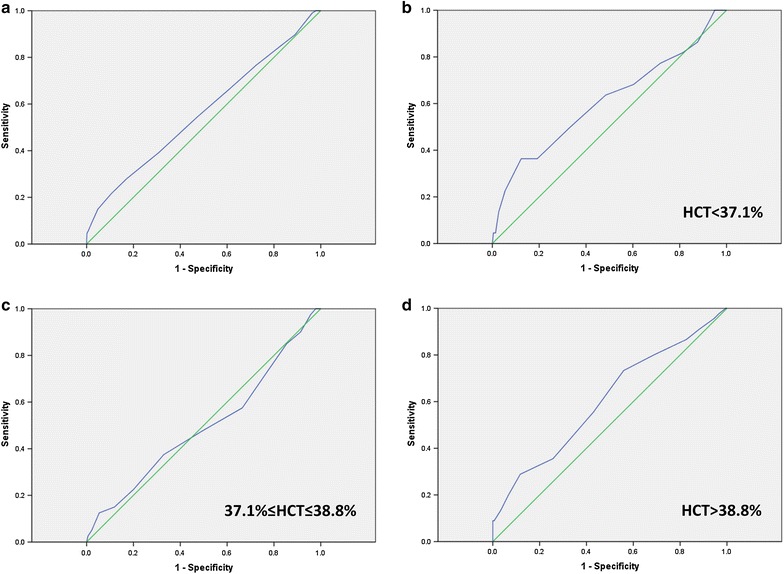
ROC curves showing the sensitivity and specificity of HbA1c alone and in combination with HCT in detecting GDM diagnosed by the IADPSG criteria. **a** HbA1c alone. **b** Combined with HCT < 37.1%; **c** Combined with 37.1% ≤ HCT ≤ 38.8%. **d** Combined with HCT > 38.8%

An ROC curve (Fig. 1a) was plotted to determine the sensitivity and specificity of HbA1c at 12–16 weeks of gestation in detecting GDM. The area under the ROC curve (AUC) of HbA1c to detect GDM was 0.563 [95% CI 0.50–0.625, P 0.038], indicating that HbA1c alone is a poor test for predicting GDM. As shown in Table 2, an HbA1c cutoff value < 4.55% (26 mmol/mol) showed a sensitivity of 85.0% and a specificity of 17.0% to rule out GDM. The negative predictive value (NPV) was 86.1%, and the positive predictive value (PPV) was 15.8%. Using an HbA1c cutoff value ≥ 5.25% (34 mmol/mol) to diagnose GDM showed a specificity of 95.2% and a sensitivity of 15%. The PPV was 36.4%, and the NPV was 85.9%.

**Table 2:** The values of HbA1c to detect GDM diagnosed by IADPSG criteria

Cut point (%)	Sensitivity	Specificity	Youden index	PPV	NPV
3.45	1	0.002	0.002	0.155	1
3.75	1	0.003	0.003	0.155	1
3.95	1	0.005	0.005	0.156	1
4.05	1	0.007	0.007	0.156	1
4.15	1	0.021	0.021	0.158	1
4.25	0.991	0.036	0.027	0.159	0.954
4.35	0.963	0.058	0.021	0.159	0.895
4.45	0.897	0.11	0.007	0.156	0.853
4.55	0.85	0.17	0.02	0.158	0.861
4.65	0.766	0.276	0.043	0.163	0.866
4.75	0.664	0.391	0.055	0.167	0.864
4.85	0.542	0.53	0.072	0.175	0.863
4.95	0.393	0.691	0.084	0.189	0.861
5.05	0.28	0.828	0.109	0.231	0.862
5.15	0.215	0.895	0.11	0.274	0.861
5.25	0.15	0.952	0.102	0.364	0.859
5.35	0.084	0.981	0.065	0.45	0.854
5.45	0.056	0.993	0.049	0.6	0.851
5.55	0.047	0.998	0.045	0.833	0.85
5.65	0.019	1	0.019	1	0.847
5.8	0.009	1	0.009	1	0.846

*PPV* positive predictive value, *NPV* negative predictive value

When combining with an HCT < 37.1%, the AUC of HbA1c to detect GDM was 0.608 (95% CI 0.468, 0.748, P 0.095) and combined with 37.1% ≤ HCT ≤ 38.8%, the AUC of HbA1c to detect GDM was 0.497 (95%CI 0.392, 0.601, P 0.948), which both indicating that HbA1c cannot be used to diagnose GDM (Fig. 1b, c). When combined with an HCT > 38.8%, the AUC of HbA1c to detect GDM was 0.605 (95% CI 0.509, 0.701], P 0.03), indicating that HbA1c is a better test for predicting GDM (Fig. 1d).

As shown in Table 3, combined with HCT > 38.8%, an HbA1c cutoff value < 4.55% (26 mmol/mol) showed a sensitivity of 86.7% and a specificity of 17.3% to rule out GDM. The NPV was 83.8%, and the PPV was 20.9%. Using an HbA1c cutoff value of 4.75% (28 mmol/mol) showed a sensitivity of 73.3% and a specificity of 44.1% to rule out GDM. The NPV was 86.8%, and the PPV was 24.8%. The Youden index was highest at 0.175. Using an HbA1c cutoff value ≥ 5.25% (34 mmol/mol) to diagnose GDM showed a specificity of 96.6% and a sensitivity of 13.3%. The PPV was 50%, and the NPV was 81.6%.

**Table 3:** The values of HbA1c combined with HCT to detect GDM diagnosed

Cut point (%)	Sensitivity	Specificity	Youden index	PPV	NPV
4.25	0.978	0.034	0.011	0.203	0.857
4.35	0.956	0.056	0.011	0.203	0.833
4.45	0.911	0.117	0.028	0.206	0.84
4.55	0.867	0.173	0.04	0.209	0.838
4.65	0.8	0.313	0.113	0.226	0.862
4.75	0.733	0.441	0.175	0.248	0.868
4.85	0.556	0.57	0.125	0.245	0.836
4.95	0.356	0.743	0.099	0.258	0.821
5.05	0.289	0.883	0.172	0.382	0.832
5.15	0.2	0.933	0.133	0.429	0.823
5.25	0.133	0.966	0.1	0.5	0.816
5.35	0.089	0.994	0.083	0.8	0.813
5.5	0.089	1	0.089	1	0.814
5.65	0.044	1	0.044	1	0.806
5.8	0.022	1	0.022	1	0.803

*PPV* positive predictive value, *NPV* negative predictive value

## Discussion

GDM is characterized by the impairment of first-phase insulin secretion function and insulin resistance. Additionally, insulin resistance gradually increases with gestational age. In this study, we found that the combined use of HbA1c and HCT compensated for the lack of sensitivity and specificity in HbA1c alone.

In agreement with various international guidelines [9, 19], HbA1c has been increasingly used as a diagnostic criterion for diabetes with a cut-off point of 6.5% in non-pregnant persons. Although HbA1c is not currently recommended for the diagnosis of GDM [7], there are several studies that advocate HbA1c as a screening test for undiagnosed GDM [20, 21]. The usefulness of HbA1c as a tool to assess the glycaemic status in pregnant women remains controversial, and currently the normal ranges for HbA1c during pregnancy are not well defined. This is because HbA1c is influenced by different factors, such as anaemia, less haemoglobin glycosylation in the first trimester, increased red cell turnover, physiological hydremia in pregnancy, slower intestinal passage, and nutritional changes [22]. In a retrospective study, an HbA1c cutoff value < 4.8% (29 mmol/mol) showed adequate sensitivity to exclude GDM (85.0%) but low specificity (31.8%), while an HbA1c cutoff value of 5.5% (37 mmol/mol) presented adequate specificity (95.7%) but low sensitivity (14.8%) in diagnosing GDM [23]. Moreover, another report showed an HbA1c value < 5.5% to rule out if GDM had a sensitivity of 82.1%, and an HbA1c value of 7.5% to diagnose GDM had a specificity of 95.8% [24].

To compensate for the lack of sensitivity and specificity in HbA1c alone, we found that HCT may be a good marker because HCT showed a significant positive correlation with homeostasis model assessment insulin resistance (HOMA-IR) [25], and HOMA-IR increased across quartiles of HCT after adjustment for age, gender, ethnicity, and smoking [16]. These findings suggest that clinically relevant haematological variables may be related to the underlying pathophysiological changes associated with diabetes. Although the pathogenesis between HCT and the development of GDM is still not clearly understood, HCT was higher in the GDM population [15].

There is a different sensitivity and specificity among the cutoff values of HbA1c in our study compared to those of previous studies. Our study also showed that the combined use of HbA1c and HCT compensated for the lack of sensitivity in HbA1c alone when combined with HCT < 37% and 37.1% ≤ HCT ≤ 38.8%, indicating that HbA1c cannot predict GDM. When combined with an HCT > 38.8%, HbA1c is a good test for predicting GDM in early pregnancy stage. HOMA-IR was positive that HCT contributes to the risk of developing type 2 diabetes [26]; thus, we thought the dilution of blood may be a protective factor for GDM. A report indicated that using an HbA1c level of 5.4% (36 mmol/mol) in the third trimester (26 weeks) has a specificity of 95% and an NPV of 91% in detecting GDM. The low sensitivity of 27% of HbA1c becomes a hurdle in standardizing such a test in pregnancy [27], and women may not benefit from early screening in pregnancy. Another report showed that an HbA1c level of 5.7–6.4% (39–46 mmol/mol), which was performed at ≤ 20 weeks of gestation, is an effective method for identifying patients ata highest risk of developing GDM; However, the suggested HbA1c values could not be used as a screening test among non-obese women because in this subgroup of the cohort, the GDM prevalence was not significantly different between the subjects with HbA1c levels of 5.7–6.4% and < 5.7% [28]. Likewise, a report indicated that women with high risk factors, such as BMI, who develop GDM have higher first trimester HbA1c values. Values above 6.0% (42 mmol/mol) are predictive of GDM, but the main limitations of their study were retrospective and they have no information on the HbA1c values of women at a low risk for GDM [22]. Moreover, in a smaller retrospective study of 145 high-risk Saudi Arabian women, the use of HbA1c at 6% (42 mmol/mol) could detect 87% of the patients with GDM diagnosed through OGTT. However, they missed 12% of the diagnoses [29]. A meta-analysis of 43 studies [30], involving over 2812 patients with GDM in China compared with 5918 controls, concluded that based on the summary ROC analysis, HbA1c is a useful diagnostic tool for confirming GDM. The authors recommended HbA1c to be tested in parallel with conventional tests; however, the meta-analysis was performed to establish the overall accuracy of HBA1c for the diagnosis of Chinese patients with GDM from 24 to 28 weeks, which was apparently different from pregnancy weeks of our study.

The Youden index, a main summary index for the receiver operating characteristic (ROC) curve, is a comprehensive measurement for the effectiveness of a diagnostic test. For a continuous-scale diagnostic test, the optimal cutoff point for positive disease is the cutoff point leading to the maximization of the sum of sensitivity and specificity [18]. Finding the Youden index of the test is equivalent to maximizing the sum of sensitivity and specificity for all the possible values of the cutoff point, but interestingly, when combining HbA1c with HCT (> 38.8%) for the screening for GDM, an HbA1c cutoff value of 4.75% presented the highest Youden index of 0.175, with the indicated specificity (73.3%) and sensitivity (44.1%) for diagnosing GDM. Compared with an HbA1c cutoff value of 4.75%, which will increase the probability of missing 13.3% of the GDM patients, and compared with an HbA1c cutoff value ≥ 5.25%, approximately 25.2% of patients are wrongly diagnosed. Therefore, we thought the cutoff point based on the Youden index was not the best clinical choice in our cohort. Moreover, it seems that anther choice was an HbA1c cutoff value < 4.25%, which showed a sensitivity of 97.8%, but only 3% of women were enrolled, while an HbA1c cutoff value of 5.50% to diagnose GDM showed a specificity of 100%, but only 1% GDM of women were enrolled. Our aim was to find a useful tool to predict the GDM for early management. We thought that an HbA1c cutoff value < 4.55% and an HbA1c cutoff value ≥ 5.25% would be more meaningful.

Some of the limitations of this study were that it was not representative of the general population, which may be related to genetic differences in the concentration of haemoglobin, the rates of glycation, and the lifespan or amount of red blood cells. Other limitations are that though the negative predictive value of HbA1c seems significant, we cannot use to exclude GDM. The HbA1c cutoff value < 4.15% showed the highest negative predictive value of 100%, but only 5.3% of the women were enrolled. When combined with HCT > 38.8%, the HbA1c cutoff value < 4.25% showed a negative predictive value of 85.7%, but only 3.1% of women were enrolled. Therefore, there are not sufficient data to give a cutoff value of HbA1c for excluding GDM. Therefore, a large-scale study in various populations in China should be conducted.

In conclusion, using ROC analysis, an HbA1c threshold ≥ 5.25% (34 mmol/L) performs better in predicting GDM when combined with an HCT > 38.8%. The combined use of HbA1c and HCT might be a more sensitive and specific screening tool for the early identification of individuals with GDM.
